# Predictors of Indoor Radon Concentrations in Pennsylvania, 1989–2013

**DOI:** 10.1289/ehp.1409014

**Published:** 2015-04-09

**Authors:** Joan A. Casey, Elizabeth L. Ogburn, Sara G. Rasmussen, Jennifer K. Irving, Jonathan Pollak, Paul A. Locke, Brian S. Schwartz

**Affiliations:** 1 Department of Environmental Health Sciences, and; 2 Department of Biostatistics, Johns Hopkins Bloomberg School of Public Health, Baltimore, Maryland, USA; 3 Center for Health Research, Geisinger Health System, Danville, Pennsylvania, USA; 4 Department of Health Policy and Management, Johns Hopkins Bloomberg School of Public Health, Baltimore, Maryland, USA; 5 Department of Medicine, Johns Hopkins School of Medicine, Baltimore, Maryland, USA

## Abstract

**Background:**

Radon is the second-leading cause of lung cancer worldwide. Most indoor exposure occurs by diffusion of soil gas. Radon is also found in well water, natural gas, and ambient air. Pennsylvania has high indoor radon concentrations; buildings are often tested during real estate transactions, with results reported to the Department of Environmental Protection (PADEP).

**Objectives:**

We evaluated predictors of indoor radon concentrations.

**Methods:**

Using first-floor and basement indoor radon results reported to the PADEP between 1987 and 2013, we evaluated associations of radon concentrations (natural log transformed) with geology, water source, building characteristics, season, weather, community socioeconomic status, community type, and unconventional natural gas development measures based on drilled and producing wells.

**Results:**

Primary analysis included 866,735 first measurements by building, with the large majority from homes. The geologic rock layer on which the building sat was strongly associated with radon concentration (e.g., Axemann Formation, median = 365 Bq/m^3^, IQR = 167–679 vs. Stockton Formation, median = 93 Bq/m^3^, IQR = 52–178). In adjusted analysis, buildings using well water had 21% higher concentrations (β = 0.191, 95% CI: 0.184, 0.198). Buildings in cities (vs. townships) had lower concentrations (β = –0.323, 95% CI: –0.333, –0.314). When we included multiple tests per building, concentrations declined with repeated measurements over time. Between 2005 and 2013, 7,469 unconventional wells were drilled in Pennsylvania. Basement radon concentrations fluctuated between 1987 and 2003, but began an upward trend from 2004 to 2012 in all county categories (*p* < 0.001), with higher levels in counties having ≥ 100 drilled wells versus counties with none, and with highest levels in the Reading Prong.

**Conclusions:**

Geologic unit, well water, community, weather, and unconventional natural gas development were associated with indoor radon concentrations. Future studies should include direct environmental measurement of radon, as well as building features unavailable for this analysis.

**Citation:**

Casey JA, Ogburn EL, Rasmussen SG, Irving JK, Pollak J, Locke PA, Schwartz BS. 2015. Predictors of indoor radon concentrations in Pennsylvania, 1989–2013. Environ Health Perspect 123:1130–1137; http://dx.doi.org/10.1289/ehp.1409014

## Introduction

Exposure to radon-222—an inert, odorless, and carcinogenic gas—is the second leading cause of lung cancer worldwide (Darby et al. 2005; Pawel and Puskin 2004). The U.S. Environmental Protection Agency (EPA) estimates that indoor radon exposure causes or contributes to about 21,000 lung cancer deaths in the United States annually (Pawel and Puskin 2004). In 1986, the U.S. EPA set an action level of 148 Bq/m^3^ (4 pCi/L; there are 37 Bq/m^3^ per pCi/L) based on the current state of radon testing and mitigation technologies [National Research Council (NRC) 1999a; U.S. EPA 1992].

Uranium-238 occurs naturally in soil and bedrock and decays to radium-226, which decays to radon. Both uranium-238 and radium-226 persist in the environment (half-lives of 4.5 billion years and 1,600 years, respectively). Radon-222 has a half-life of 3.8 days, and its radioactive decay products are responsible for its carcinogenicity. Pressure differentials between soil gas and indoor air cause the migration of radon through cracks and other openings into buildings, the primary source of indoor radon. Radium and radon are soluble in water, with concentrations increasing as salinity increases (Warner et al. 2012).

Several counties in eastern Pennsylvania overlie the Reading Prong, a physiographic section known to have high bedrock uranium concentrations (Gundersen 1991) and elevated indoor radon levels. The entire state has had some of the highest indoor radon levels in the United States. The Pennsylvania Department of Environmental Protection (PADEP) established a Radon Division that administers a program of radon monitoring and remediation (http://www.portal.state.pa.us/portal/server.pt/community/radon_division/21923).

U.S. Geological Survey (USGS) analysis of 548,547 indoor and short-term radon test results compiled by the PADEP during 1990–2007 reported that 39% of radon tests exceeded the U.S. EPA action level and that concentrations varied dramatically by geologic unit, a rock layer of a given lithology and geologic period (e.g., Annville Formation, high-calcium limestone from the Ordovician period) (Gross 2013). Geologists have identified 195 geologic units in Pennsylvania. Other factors that have been associated with higher indoor radon levels include the use of radon-rich well water [Folger et al. 1994; United Nations Scientific Committee on the Effects of Atomic Radiation (UNSCEAR) 2009], colder months, less precipitation, more expensive housing, rural area, and higher individual socioeconomic status (SES) (Cohen and Gromicko 1988; Folger et al. 1994; UNSCEAR 2009). Radon is present in natural gas used for cooking and heating; calculations performed in the 1970s suggested that it would not be expected to result in an increase in indoor radon levels (Johnson et al. 1973). Radon can also enter buildings from ambient air; however, outdoor radon concentrations are generally low, around 10 Bq/m^3^, but can range from 1 to 100 Bq/m^3^ (UNSCEAR 2009).

The development of unconventional natural gas in the Marcellus shale in Pennsylvania has the potential to exacerbate several pathways for entry of radon into buildings. The USGS reported 91,020 Bq/m^3^ (*n* = 14) as the median radium concentration in produced water from Marcellus wells (Rowan et al. 2011), a value nearly 500 times the federal drinking water limit (185 Bq/m^3^) and one that far exceeds the industrial discharge limit of 2,220 Bq/m^3^. Underground, radon collects in porous geological formations and thus in natural gas production wells (Gogolak 1980). Shales also tend to contain both higher concentrations of uranium (3.7–40 ppm) than other geologic formations and higher concentrations of radon in their natural gas (Gogolak 1980). The USGS reported preliminary data from 11 wellheads in Pennsylvania with corrected concentrations of radon (devices were calibrated for air measurement, but used in natural gas with correction factor = gas measurement × 1.47) ranging from 37 to 2,923 Bq/m^3^ (median = 1,369) (Rowan and Kraemer 2012), suggesting that shale gas may have higher radon levels than other natural gas sources.

To our knowledge, no prior studies have evaluated predictors of radon concentrations in Pennsylvania. Our main objective was to identify the independent contribution to indoor radon concentrations of geologic unit, water source, building characteristics, season, weather, community SES, community type, and Marcellus shale development.

## Methods

**Figure 1:**
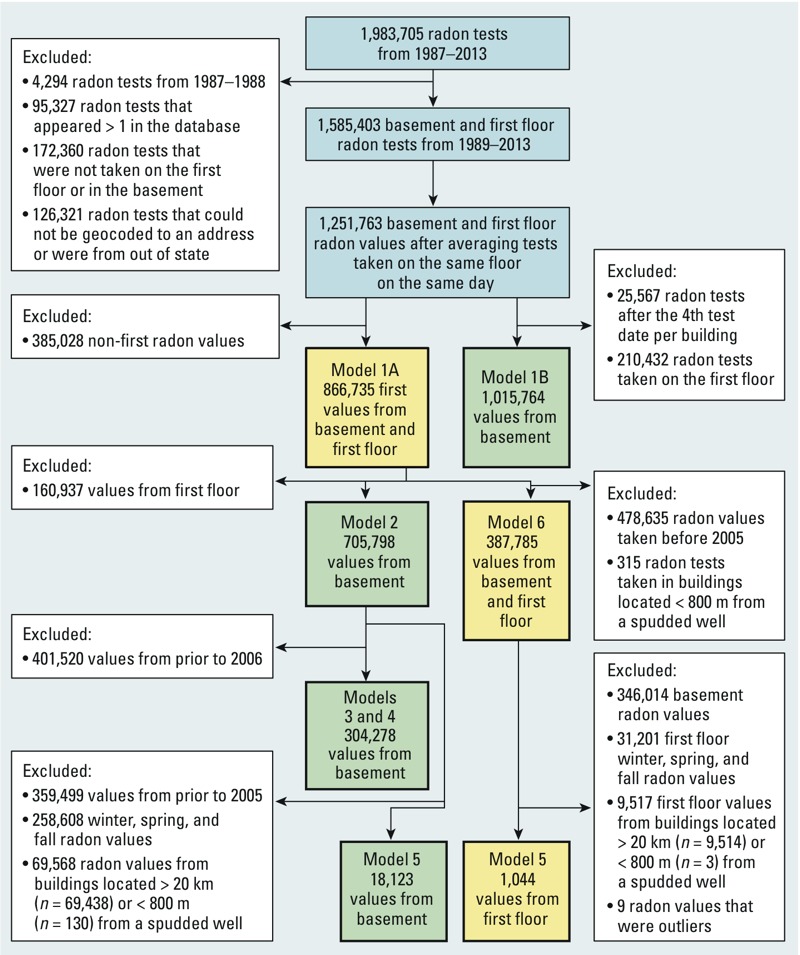
Flow diagram of radon tests included in six primary models.

*Study design*. We obtained radon data on 1,983,705 indoor radon tests conducted in 806,469 buildings between 1987 and 2013 from all 67 counties in Pennsylvania; these tests were submitted by certified testers, laboratories, or homeowners to the PADEP Bureau of Radiation Protection, Radon Division. Buildings are most often tested during real estate transactions (World Health Organization 2009), and the PADEP requires reporting of all test results to their GreenPort website (http://www.depgreenport.state.pa.us/). We used the subset of radon measurements taken between 1 January 1989 and 31 December 2013 in our analysis because few samples were available from earlier years (*n* = 4,294) (Figure 1). The Institutional Review Board at the Johns Hopkins Bloomberg School of Public Health reviewed the study protocol and did not consider it to be human subjects research.

*Outcome: indoor radon concentration*. Data included the address of the tested building, building type (12 types; Table 1), test location (i.e., basement, first floor, second floor), test type (i.e., activated charcoal, alpha-track detectors, charcoal liquid scintillation, continuous radon monitors, electret ion chamber), test dates, and radon concentration (Bq/m^3^). Results were available for both short-term (2–7 days) and long-term (up to 1 year) testing periods. We used ArcGIS (version 10.0; Esri) and 10 street maps [e.g., TeleAtlas (TomTom), TIGER files (https://www.census.gov/geo/maps-data/data/tiger.html), and StreetMap Premium (Esri) from 2000–2012] to obtain latitude and longitude of buildings.

**Table 1:** Radon concentrations (Bq/m^3^) by test and building characteristics, stratified by test location.

Variable	Category	Basement*			First floor*		
		*n* (%)	Median (IQR)	Range	*n* (%)	Median (IQR)	Range
All results	Total	705,798 (100)	118.4 (59.2–262.7)	0–69,057	160,937 (100)	55.5 (29.6–111.0)	0–111,481
U.S. EPA action level	< 148 Bq/m^3^	408,184 (57.8)	66.6 (40.7–99.9)	0–147.9	131,245 (81.6)	44.4 (25.9–74)	0–147.9
	≥ 148 Bq/m^3^	297,614 (42.2)	310.8 (207.2–580.9)	148–69,057	29,692 (18.5)	262.7 (188.7–447.7)	148–111,481
Well water use	No	591,565 (83.8)	111.0 (58.1–236.8)	0–69,057	138,804 (86.3)	51.8 (27.8–103.6)	0–111,481
	Yes	114,233 (16.2)	185.0 (81.4–458.8)	0–55,463	22,133 (13.8)	74.0 (37.0–164.7)	0–14,822
Building type	2-Story	298,672 (42.3)	114.7 (61.1–114.7)	0–55,463	73,340 (45.6)	53.65 (29.6–103.6)	0–111,481
	3-Story	69,008 (9.8)	166.5 (77.6–166.5)	2.2–33,973	8,837 (5.5)	70.3 (33.3–162.8)	0.7–7,478
	Apartment	1,999 (0.3)	82.0 (44.4–173.9)	3.7–5,254	1,042 (0.7)	33.3 (18.5–68.5)	0.7–1,395
	Bi-level	12,599 (1.8)	131.3 (62.9–294.2)	1.1–25,937	2,628 (1.6)	77.7 (37.0–166.5)	1.9–9,476
	Cape Cod	15,801 (2.2)	127.7 (70.3–257.2)	0–29,637	3,837 (2.4)	59.2 (29.6–103.6)	0–29,711
	Commercial	1,773 (0.3)	77.7 (42.6–157.3)	3.7–4,449	871 (0.5)	40.7 (22.2–83.9)	0.7–6,915
	Contemporary	4,156 (0.6)	136.0 (66.6–296)	3.7–25,530	1,968 (1.2)	51.8 (25.9–108.8)	3.7–2,760
	Public/school	370 (0.1)	94.7 (47.2–203.5)	13.6–5,176	202 (0.1)	51.8 (27.1–96.2)	3.7–636
	Ranch	63,946 (9.1)	151.7 (79.6–323.2)	0.9–69,057	14,764 (9.2)	66.6 (37.0–136.9)	0–10,286
	Split level	17,788 (2.5)	107.3 (59.2–218.3)	1.5–41,607	5,822 (3.6)	59.2 (33.3–107.3)	0–8,251
	Townhouse	42,691 (6.1)	68.5 (40.7–125.8)	0.2–32,751	16,920 (10.5)	37.0 (22.2–66.6)	0–22,459
	Trailer	183 (0.03)	88.8 (51.8–192.4)	18.5–2,531	139 (0.1)	33.3 (18.5–33.3)	3.7–662
	Unknown	176,812 (25.1)	122.1 (55.5–297.9)	0–35,668	30,567 (19.0)	62.9 (29.6–153.0)	0–16,119
Test type	Activated charcoal	237,932 (33.7)	129.5 (55.5–325.6)	0–69,057	54,957 (34.2)	59.2 (25.9–142.5)	0–50,294
	Alpha track	7,074 (1.0)	161.1 (81.4–333.0)	0.7–14,796	1,844 (1.2)	99.9 (42.7–221.4)	0.4–3,441
	Charcoal liquid scintillation	44,936 (6.4)	162.8 (70.3–392.2)	0–32,751	4,934 (3.1)	77.7 (33.3–186.9)	3.7–16,119
	Continuous	209,994 (29.8)	114.7 (59.2–236.8)	0.2–41,544	14,647 (9.1)	48.1 (25.9–92.5)	0.1–111,481
	Electret ion chamber	205,862 (29.2)	111.0 (62.9–214.6)	0–62,974	84,555 (52.5)	53.65 (29.6–99.9)	0–29,711
Test duration	1–7 days	693,864 (98.3)	118.4 (59.2–262.7)	0–69,057	157,912 (98.2)	55.5 (29.6–111.0)	0–111,481
	≥ 8 days	11,934 (1.7)	148.0 (74.0–310.8)	0–69,057	3,025 (1.8)	81.4 (37.0–181.3)	0–3,593
Season	Winter	169,921 (24.1)	114.7 (59.2–247.9)	0–55,463	37,886 (23.5)	48.1 (25.9–96.2)	0–50,294
	Spring	198,485 (28.1)	114.7 (59.2–229.4)	0–62,974	46,432 (28.9)	51.8 (27.8–98.1)	0–22,496
	Summer	174,007 (24.7)	133.2 (66.6–299.7)	0–41543.6	40,320 (25.1)	66.6 (33.3–136.9)	0–111,481
	Autumn	163,385 (23.2)	118.4 (59.2–292.3)	0–69,057	36,886 (22.6)	59.2 (29.2–129.5)	0–29,711
Average temperature in month of test (°C)	< 0	84,259 (11.9)	3.3 (1.6–8.0)	0.004–930	17,294 (10.8)	1.6 (0.8–3.7)	0–276
	0 to < 10	232,372 (32.9)	3.3 (1.6–7.7)	0–1,866	53,651 (33.3)	1.6 (0.8–3.4)	0–3,013
	10 to < 18.3	189,693 (26.9)	3.4 (1.7–7.3)	0–1,499	43,018 (26.7)	1.5 (0.8–3.0)	0–607
	≥ 18.3	199,474 (28.3)	3.0 (1.6–6.2)	0–1,702	46,974 (29.2)	1.4 (0.7–2.6)	0–608
Average rainfall in month of test (cm)	< 7.1	236,239	125.8 (62.9–281.9)	0–69,056	53,693	59.2 (29.6–121.0)	0–50,294
	7.2–10.8	232,866	116.6 (59.2–251.6)	0–62,974	55,928	55.5 (29.6–107.3)	0–111,481
	≥ 10.9	236,693	116.6 (59.2–255.3)	0–41,607	51,316	53.7 (27.8–107.3)	0–22,496
Community socioeconomic deprivation quartile^*a*^	1 (< –4.9)	169,327 (24.5)	118.4 (59.2–262.7)	0–35,897	46,100 (29.4)	59.2 (29.6–118.4)	0–50,294
	2 (–4.9 to –3.3)	172,068 (24.9)	133.2 (66.6–306.0)	0–55,463	37,389 (23.8)	62.9 (33.3–129.5)	0–29,711
	3 (–3.2 to –1.1)	177,619 (25.7)	129.5 (66.6–284.9)	0–69,057	36,734 (23.4)	59.2 (29.6–114.7)	0–18,537
	4 (≥ –1.0)	173,407 (25.0)	103.6 (53.7–222.0)	0–35,668	36,742 (23.4)	44.4 (24.1–92.5)	0–111,481
Minor civil division	Township	488,168 (69.2)	130.7 (64.8–299.7)	0–55,463	116,311 (72.3)	59.2 (29.6–122.1)	0–50,294
	Borough	133,990 (19.0)	112.9 (59.2–233.1)	0–69,057	25,643 (15.9)	51.8 (25.9–103.6)	0–22,496
	City	83,638 (11.9)	79.6 (44.4–148.0)	0–31,361	18,983 (11.8)	40.7 (22.2–70.7)	0–111,481
County category^*b*^
No Marcellus activity	Other counties	379,223 (53.7)	120.3 (59.2–273.8)	0–62,974	112,252 (69.8)	55.5 (29.6–111.0)	0–50,294
Low Marcellus activity	< 100 drilled wells by 2013	174,216 (24.7)	114.7 (62.9–233.1)	0–30,621	22,734 (14.1)	55.5 (27.4–118.4)	0–22,496
High Marcellus activity	≥ 100 drilled wells by 2013	57,814 (8.2)	129.5 (70.3–260.9)	0–30,858	5,753 (3.6)	62.9 (33.3–129.5)	2.6–111,481
Reading Prong	Berks, Lehigh, and Northampton	62,635 (8.9)	192.4 (85.1–425.5)	0–69,057	9,632 (6.0)	96.2 (44.4–210.9)	0–14,822
Philadelphia	Philadelphia	31,910 (4.5)	62.9 (37.0–105.5)	0–31,361	10,566 (6.6)	37.0 (22.2–62.9)	0–2,331
Drilled well within 20 km of building	No	637,317 (90.3)	118.4 (59.2–266.4)	0–69,057	156,731 (97.4)	55.5 (29.6–111.0)	0–50,294
	Yes	68,481 (9.7)	124.0 (70.3–244.2)	0–38,658	4,206 (2.6)	59.2 (33.3–120.3)	3.7–111,481
Drilled-well exposure quartile^*c*^
1	< 0.19 well/km^2^	17,086 (25.0)	120.3 (70.3–225.7)	3.7–23,465	1,086 (25.8)	70.3 (37.0–133.2)	3.7–2,742
2	0.19 to 0.61 well/km^2^	17,099 (25.0)	125.8 (70.3–255.3)	18.5–29,637	1,073 (25.5)	59.2 (29.6–114.7)	18.5–8,251
3	0.62 to 1.4 well/km^2^	17,126 (25.0)	125.8 (70.3–247.9)	18.5–30,858	1,046 (24.9)	55.5 (29.6–107.3)	14.8–3,559
4	> 1.4 well/km^2^	17,170 (25.1)	125.8 (70.3–247.9)	18.5–19,769	1,001 (23.8)	59.2 (37.0–122.1)	18.5–111,481
Producing-well exposure quartile^*d*^
1	< 2.55 m^3^/day/km^2^	83,971 (24.3)	111.0 (55.5–247.9)	2.6–40,928	13,052 (31.2)	51.8 (26.3–99.9)	0–8,131
2	2.55 to 294.4 m^3^/day/km^2^	86,196 (24.9)	120.3 (61.1–266.4)	7.4–35,897	10,826 (25.9)	59.2 (30.9–118.4)	0–29,711
3	294.5 to 4312.6 m^3^/day/km^2^	86,989 (25.1)	125.8 (62.9–281.2)	11.1–62,974	10,034 (24.0)	59.2 (33.3–122.1)	3.7–12,119
4	> 4312.7 m^3^/day/km^2^	89,143 (25.7)	133.2 (70.3–288.6)	11.1–30,858	7,879 (18.9)	61.1 (33.3–124.0)	5.6–111,481
^***a***^Not available for buildings located in Philadelphia or Pittsburgh. Community socioeconomic deprivation was assigned at the township, borough, or census-tract level, based on six indicators derived from the 2000 U.S. Census: combined less than high school education, not in the labor force, in poverty, on public assistance, civilian unemployment, and does not own a car; a higher score represents a more deprived community. ^***b***^No Marcellus activity (other counties): Adams, Bedford, Bucks, Carbon, Chester, Cumberland, Dauphin, Delaware, Erie, Franklin, Fulton, Juniata, Lancaster, Lebanon, Mifflin, Montgomery, Montour, Northumberland, Perry, Pike, Schuylkill, Snyder, Union, and York; low Marcellus activity counties: Allegany, Beaver, Blair, Cambria, Cameron, Centre, Clarion, Columbia, Crawford, Elk, Forest, Huntingdon, Indiana, Jefferson, Lackawanna, Lawrence, Luzerne, McKean, Mercer, Potter, Somerset, Sullivan, Venango, Warren, and Wayne; high Marcellus activity counties: Armstrong, Bradford, Butler, Clinton, Clearfield, Fayette, Greene, Lycoming, Susquehanna, Tioga, Washington, Westmoreland, and Wyoming. ^***c***^Restricted to 2005–2013 and buildings within 20 km of a drilled well at the time of the radon test. ^***d***^Restricted to 2005–2013. *Categories of all variables shown had statistically significantly different ln-radon concentrations by ANOVA.							

We excluded tests from buildings that could not be geocoded to an address, that were out of state, that were not taken on the first floor or basement, or that appeared in the database more than once (*n* = 394,008 buildings). Many buildings (*n* = 307,245) had multiple radon measurements (range, 2–56) taken on the same floor and day. For example, in buildings with two measurements per floor (*n* = 291,098), the correlation of floor-specific measurements was very high (ρ = 0.91). Because we had no information on building remediation, our primary analysis included only measurements taken during the first test day at each building (*n* = 866,735, including 224,666 averaged concentrations from the same floor and day). In a sensitivity analysis, we included up to four tests over time from each building.

**Figure 2:**
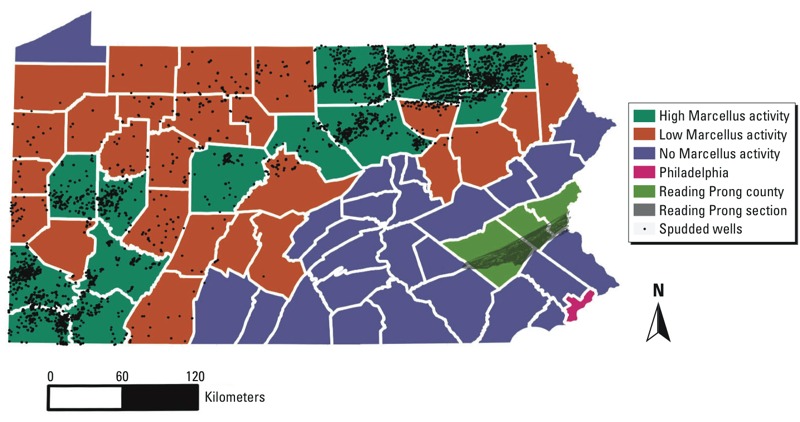
County category groupings, the Reading Prong section, and location of spudded Marcellus wells (through 2013).

*Data sources*. We obtained data on the public water service areas compiled by the PADEP from the Pennsylvania State University’s Spatial Data Access website (Pennsylvania Spatial Data Access; http://www.pasda.psu.edu). Any home outside the public water supplier’s service area was assumed to use well water. Statewide bedrock geology and physiographic sections were downloaded as shapefiles from the Pennsylvania Department of Conservation and Natural Resources (PADCNR; http://www.dcnr.state.pa.us). On average, each geologic unit covers 749 noncontiguous square kilometers. One important geologic unit is the felsic gneiss, which is found throughout the state. The Reading Prong section primarily contains felsic gneiss; however, the section is present in only three counties, identified as Reading Prong counties in Figure 2.

We downloaded monthly average temperature and rainfall in 10 regions from the Pennsylvania State Climatologist (http://climate.psu.edu). Based on 2000 U.S. Census boundary files, buildings were assigned to a minor civil division: cities, moderate- to high-density boroughs, and suburban and rural townships. Community socioeconomic deprivation, an indicator of community SES, was derived from six *z*-transformed U.S. Census 2000 variables (Schwartz et al. 2011). Marcellus shale development data covering 1 January 2005 through 31 December 2013, came from PADEP and PADCNR, with the latitude and longitude of each well, the date of well drilling, natural gas produced, and number of producing days.

**Figure 3:**
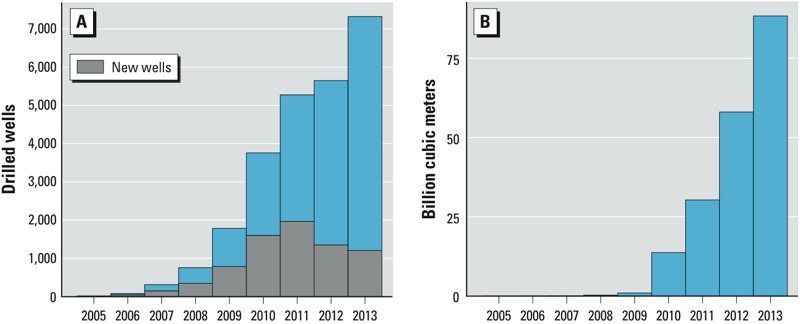
(*A*) Number of new unconventional wells drilled annually during 2005–2013 (gray) and cumulative number of wells. (*B*) Unconventional natural gas produced (billions of cubic meters) during 2005–2013.

*Marcellus shale development metrics*. The Marcellus Formation is 1,500–2,500 m underground and underlies a large section of Pennsylvania from the southwest curling northeast. Only unconventional wells (horizontal wells, hydraulic fracturing) were included (Figures 2 and 3).

The spud date was the day well drilling began, and the production start date was the day the well first produced natural gas. We estimated a start date of production for each well:

Production start date at well *i* = (*l_p_* – *k_p_*)*I_p_*, [1]

where *l_p_* is the last day of production in period *p*, *k_p_* is the number of days in production in period *p*, and *I_p_* equals 1 when period *p* is the first period of production for well *i*, and 0 otherwise. We estimated daily natural gas production for each well in its first production period as the volume of gas produced in its first period divided by the number of days of reported production. In subsequent periods we estimated daily gas production as the volume of gas reported in each period, divided by the number of days in that production period. When wells were missing one or more production volumes by period, we imputed missing volumes for periods in which there were data before and after (*n* = 102 wells), assuming a linear decline over time. We imputed missing spud dates (*n* = 149 wells) using conditional mean imputation based on production start date, stimulation (hydraulic fracturing) date, year, and geographic region.

Two primary Marcellus development metrics were created based on all wells in the state, one on drilled wells and the other on wells in production. Wells drilled prior to the start of an indoor radon test were included in that building’s exposure assignment. Once a well was drilled, it was assumed to contribute until the end of the study period, 31 December 2013. We calculated drilled-well exposure assignment:

Building *j* metric = Σ*^^n^^_i =_*_1_Σ*^^l^^_k =_*_1_(*I_A_*(*k,m*)*/d_ij_*^2^)*/m*, [2]

where *n* is the number of drilled wells, *m* is the duration of the indoor radon test in days, *k* is the day with 1 equal to 1 January 2005, and *l* is equal to 3,287 (to 31 December 2013), *I_A_*(*k,m*) is 1 when well *i* has been drilled before day *k* and the indoor radon test at building *j* is conducted from day *k* to day *k* + *m*, and 0 otherwise, and d^2^*_ij_* is the squared-distance between the coordinates of the wellhead of well *i* and building *j*. We calculated the producing-well exposure assignment:

Building *j* metric = Σ*^^n^^_i =_*_1_Σ*^^l^^_k =_*_1_(*I_A_*(*k,m*)*g_p_/d_ij_*^2^)*/m*, [3]

where *n* is the number of producing wells, *m* is the duration of the indoor radon test in days, *k* is the day with 1 equal to 1 January 2005 and *l* is equal to 3,287 (to 31 December 2013), *I_A_*(*k,m*) is 1 when well *i* is producing on day *k* and the indoor radon test at building *j* is conducted from day *k* to day *k* + *m,* and 0 otherwise, *g_p_* is the estimated amount of natural gas produced (in thousands of cubic meters) by well *i* on day *k*, and d^2^*_ij_* is the squared distance between the coordinates of the wellhead of well *i* and building *j*.

*Statistical analysis*. The goal of the analysis was to evaluate associations of year, county category, geologic unit, community type, community SES, well water use, and metrics of unconventional natural gas development with indoor radon concentrations. Building was the unit of analysis. The distribution of radon concentrations was skewed, so we used natural log-transformed radon concentration (ln-radon) as our outcome variable to improve compliance with assumptions of linear regression (i.e., homoscedasticity and normality of residuals). We used one-way analysis of variance (ANOVA) to assess unadjusted differences in indoor radon concentrations by other covariates. To evaluate associations with indoor ln-radon concentration, we used multivariable linear regression and generalized estimating equations to account for within-building correlation when models included more than one measurement per building. When beta coefficients are < 0.1, 100 × β can be interpreted as approximating the percent change in radon concentration associated with a 1-unit change in the independent variable. In models used to assess the spatial distribution of radon levels, we wanted to remove the contribution of building-related factors. Models used to assess associations of unconventional natural gas development with radon levels did not contain county, minor civil division, or community SES because of concern about overadjustment. Covariates were included in models 1–4 because of *a priori* hypotheses that they could confound the relationship between our primary variables of interest and ln-radon concentration or based on the quasi-likelihood information criterion (Hardin and Hilbe 2013).

Model 1A included only measurements taken on the first test date at each building (*n* = 762,725 buildings and *n* = 866,735 radon values), which included some averaged values when multiple tests were performed on the same floor on the same day. Model 1A was adjusted for test year (1989–2013), test location (basement or first floor), well water use (yes or no), 13 building types (including “unknown”), test type (listed above), test duration, season, weather (average temperature and rainfall for 10 regions during the month radon measurement began with linear, quadratic, and cubic terms to account for nonlinearity), minor civil division, county (*n* = 67), and 179 mutually exclusive geologic units [reference group = Stockton Formation (*n* = 62,026) plus 12 geologic units with < 20 tests]. We used model 1B to evaluate changes over time in within-building basement radon levels by estimating model 1A, restricted to basement measurements, from up to four testing dates per building (*n* = 714,097 buildings and *n* = 1,015,764 radon values). We also assessed changes in radon levels over time for buildings with high initial concentrations by restricting model 1B to buildings with initial radon concentrations ≥ 740 Bq/m^3^ (*n* = 55,161 buildings and *n* = 99,293 radon values).

In model 2, we assessed differential changes in basement radon concentration by place and time by removing county from model 1A and restricting to basement radon values (*n* = 705,798 buildings and radon values). We ran five separate regressions by county category [Philadelphia, Reading Prong (which have no Marcellus activity), low Marcellus activity (< 100 wells drilled by 2014), high Marcellus activity (≥ 100 wells drilled by 2014), and no Marcellus activity] (see Supplemental Material, Table S1). We then plotted the predicted values of the geometric mean radon concentration by county category and year; 95% confidence intervals (CIs) were estimated using the delta method (Cox 1998).

We produced two maps of statewide basement radon concentrations for 2006–2013. The first displayed median radon concentrations per geologic unit (with ≥ 10 measurements). In the second, we removed variability due to building-level factors (which could help target remediation efforts to certain locations). We did this with model 3 by regressing ln-radon on building-level factors (i.e., year, building type, test type, test duration, season, average temperature and rainfall). In model 4, we fit a linear regression of the residuals from model 3 on only geologic unit, county, and well water use (*n* = 304,278 buildings and radon values) and then used model 4 to output new predicted radon concentrations in a 500-m × 500-m grid statewide. Split samples suggested that model 4 predicted well, and residual semivariogram plots did not exhibit spatial autocorrelation.

We used models 5 and 6 to evaluate two *a priori* hypotheses of the possible contribution of unconventional natural gas development on indoor radon concentrations: *a*) Ambient air could contribute to indoor radon concentrations through the release of radon and radium in the drilling process, primarily in the summer when buildings are more likely to be open; and *b*) produced natural gas containing radon could enter building air through use of natural gas for cooking or unvented heating and, given a transit speed of about 16 km/hr in pipelines (Gogolak 1980), all buildings in the state could be affected.

In model 5, we evaluated the associations of the drilled well metric (Equation 2) with ln-radon concentration by restricting model 1A to the years 2005–2013 (primary years of Marcellus development); measurements taken only during July, August, and September; and buildings located within 20 km of a drilled well at the time of the radon test. Because summer months had little variability in temperature, we did not include temperature in model 5. We also fit model 5 separately for first floor (*n* = 1,044 buildings and radon values) and basement (*n* = 18,123 buildings and radon values) because of hypotheses about pathways of radon entry. Model 5 excluded 3 first-floor and 130 basement radon concentrations from buildings located within 800 m of a well because we did not have enough data to fit a curve for distances < 800 m and 9 first-floor radon values that were outliers (studentized residuals > 3). As a counterfactual analysis, we re-ran model 5 including buildings from 1989 through 2005 that would be located within 20 km of a Marcellus well by December 2013.

To evaluate associations of the producing-well metric (Equation 3) and ln-radon concentrations, in model 6, we restricted model 1A to the years 2005–2013 and excluded buildings located within 800 m of a producing well (*n* = 315). Because year and the production well metric were highly correlated (ρ = 0.95), the regression models could not separate their independent influence; therefore, we presented model 6 production associations as unadjusted and adjusted for year, as well as year associations unadjusted for production. Regression analysis was performed using Stata 13 (StataCorp). We tested for linear trend by year by including year as a continuous variable. Alpha was set at 95%, and statistical significance was *p* < 0.05. Exposure metric creation and radon predictions were performed using R, version 3.0.0 (R Core Team 2013) and the sp package.

## Results

Our primary analysis included 866,735 first indoor radon values from 762,725 buildings collected during 1989–2013. Every county reported results (see Supplemental Material, Table S2), with a median of 3,447 and ranging from 59 in Forest to 99,590 in Allegheny. Most (81.4%) of the values were from basements (*n* = 705,798), with a median concentration of 118.4 Bq/m^3^ [interquartile range (IQR) = 59.2–262.7]; 42.2% of these values in basements (*n* = 297,614) met or exceeded the U.S. EPA action level (Table 1). Radon concentrations varied within and between county categories across the study period, with Reading Prong counties having significantly higher and Philadelphia significantly lower radon concentrations.

In total, 7,469 unconventional natural gas wells were drilled in 39 Pennsylvania counties during 2005–2013 (Figure 3A). More than 5,000 of those wells entered production, producing 191 billion m^3^ of natural gas during 2009–2013 (Figure 3B). We identified 1,056 buildings with radon values from the first floor collected during the summer and located within 20 km of a drilled well at the time of the test. The median of the drilled well metric of these buildings was 0.6 wells/km^2^ (IQR = 0.2–1.3). The median of the producing-well metric of buildings statewide was 294 m^3^/day/km^2^ (IQR = 3–4,464). There were increasing median radon concentrations across quartiles of the production well metric for both the first floor and basement (Table 1).

In unadjusted analysis, several variables were associated with indoor radon concentrations: well water, building type, duration of test, season, weather during the test, community SES, community type, and county; geologic unit associations were strong, with large variation by unit [e.g., Axemann Formation, median = 365 Bq/m^3^ (IQR = 167–679), vs. Stockton Formation, median = 93 Bq/m^3^ (IQR = 52–178); Table 1]. Communities with lower SES had lower radon levels, but this variable was not included in subsequent models because of concerns regarding mediation (i.e., drilling improves individual SES and community SES, but richer individuals have more tightly sealed homes and higher radon concentrations).

In adjusted analysis (model 1A, *n* = 866,735 first basement and first-floor values), many variables were associated with radon concentrations. Strong associations were observed for specific geologies, for example Axemann, Bellefonte, and Nittany Formations were associated with 220–250% higher radon concentrations, compared with the Stockton Formation (see Supplemental Material, Table S2). Alpha track (generally long-term) and charcoal liquid scintillation tests were associated with 23% and 27% higher radon levels, respectively, compared with activated charcoal tests. Buildings using well water (vs. municipal water) also had 21% higher concentrations (β = 0.191; 95% CI: 0.184, 0.198). Buildings in cities versus townships were associated with lower radon levels (β = –0.323; 95% CI: –0.333, –0.314). There were nonlinear associations of rainfall and temperature; less rainfall and cooler temperatures were generally associated with higher radon concentrations. When up to four temporally ordered basement measurements per building were evaluated (model 1B, *n* = 1,015,764), we observed a significant decrease in radon concentration across tests, with a 37.1% (95% CI: 36.7, 37.3) decline from test 1 to test 2, 51.5% (95% CI: 51.1, 51.9) from test 1 to test 3, and 58.0% (95% CI: 57.4, 58.9) from test 1 to test 4 (see Supplemental Material, Table S3). Among buildings with an initial basement radon concentration ≥ 740 Bq/m^3^, we observed from the first test an 88.8% (95% CI: 88.6, 88.9) decline to the second test, and a 92.3% (95% CI: 92.1, 92.4) decline to the third test (see Supplemental Material, Table S4).

**Figure 4:**
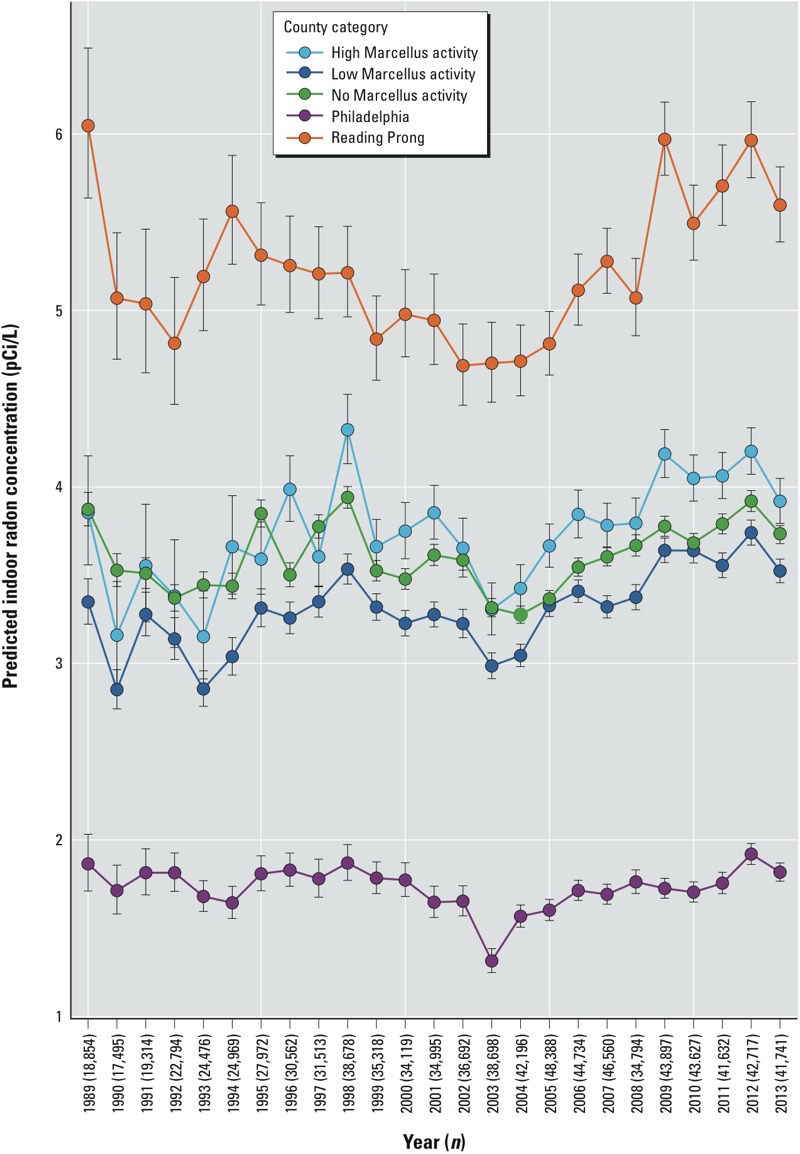
Geometric mean and 95% confidence intervals for indoor basement radon concentrations in five county categories, 1989–2013. High Marcellus shale counties had at least 100 unconventional wells drilled by 2013, and low Marcellus shale counties had 1–100. Predicted values were generated from five separate linear regression models (one for each county category) including only measurements taken on the first test date at each building (*n *= 705,798 values), adjusted for test year (1989–2013), well water use, 13 building types, five test types, test duration, season, weather (average temperature and rainfall with linear, quadratic, and cubic terms), minor civil division, and 179 mutually exclusive geologic units (model 2).

**Figure 5:**
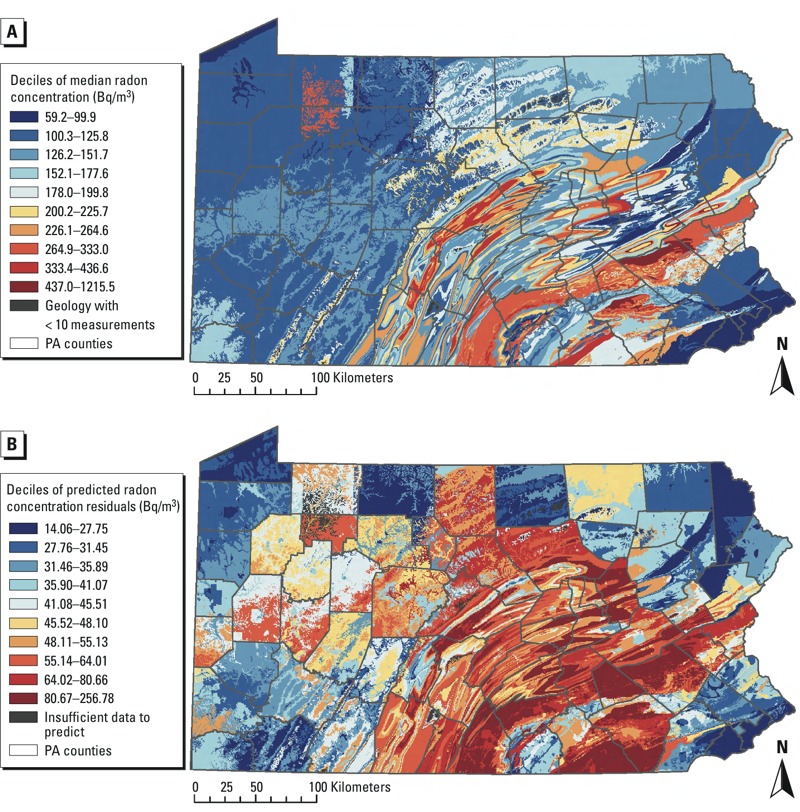
(*A*) Unadjusted median basement radon concentrations (*n *= 304,278 tests) in Pennsylvania by geologic unit during 2006–2013. (*B*) Predicted contribution to basement radon concentration from geologic unit, county, and well water after accounting for variation due to year (2006–2013), building type, test type, test duration, season, average temperature, and average rainfall (based on models 3–4; *n *= 304,278 values).

After controlling for confounding variables including geologic unit (model 2, basement values only), there was evidence of an upward trend from 2004 to 2012 (*p* < 0.001). Confidence intervals overlapped among the high, low, and no Marcellus activity counties, particularly between no activity and high activity counties before 2004, whereas there was little or no overlap after that time, with high activity counties having the highest estimated radon concentrations, followed by no activity and low activity counties, respectively. However, fewer measurements were taken in earlier years, resulting in less precise estimates with more variation from year to year (Figure 4). It should be noted that when both basement and first-floor values were included (model 1A; see Supplemental Material, Table S2) the upward trend began in 2006 (*p* < 0.001). There were large differences across the state in median radon concentrations by geologic unit (Figure 5A). Geologic unit and well water use did not appear to make large contributions to indoor radon concentrations in regions with many drilled Marcellus wells (Figure 5B, models 3 and 4).

The drilled well metric was significantly associated with first-floor summer radon concentrations in buildings located within 20 km of a drilled well; for each additional drilled well per square kilometer surrounding the building, first-floor radon levels were estimated to be 2.8% higher (drilled well β = 0.028; 95% CI: 0.001, 0.05) (model 5). We also found a positive, but attenuated, association with basement measurements (drilled well β = 0.010; 95% CI: 0.003, 0.020). In a sensitivity analysis, there was no association between the counterfactual drilled well metric for future wells and summer first-floor concentrations between 1989 and 2005 (drilled well β = 0.001; 95% CI: –0.022, 0.024).

The producing-well metric was not associated with indoor radon concentration when year was included in model 6 (production β = –0.001; 95% CI: –0.003, 0.002); when year was not associated, gas production was significantly associated with indoor radon concentration and radon concentrations were estimated to be 1.3% higher with each additional 100 m^3^ of natural gas produced per day per square kilometer (production β = 0.013; 95% CI: 0.005, 0.020). There was a positive association between year and radon concentrations between 2005 and 2013, when the production metric was removed from model 6 (year β = 0.012; 95% CI: 0.011, 0.014).

## Discussion

We identified several predictors of indoor radon concentrations in Pennsylvania, a state with historically high radon levels (Alter and Oswald 1987). Water source, building type, test type, test duration, season, weather, county, and geologic unit were associated with indoor radon concentration. When data were aggregated to county categories, on average, Reading Prong counties had the highest indoor radon concentrations (Table 1, Figure 4). Nearly 300,000 homes had a first basement test result that exceeded the U.S. EPA action level. We observed fluctuating radon concentrations throughout the study period; low Marcellus activity counties consistently had lower radon concentrations than either high or no Marcellus activity, before and after drilling began. From 2005 through 2013 the high activity counties had higher basement radon levels than either low or no Marcellus activity counties, with confidence intervals that did not overlap, and there was evidence of a significant upward trend (Figure 4). In a model using first-floor and basement values and adjusting for each county (model 1A), radon concentrations only began increasing in 2006 (see Supplemental Material, Table S2). When we included multiple basement measurements per building, radon levels declined with repeated measurements within a building, which is good news for public health and also suggests that state remediation programs are effective.

Buildings located in cities had nearly 27% lower radon levels than those located in more rural townships (Table 2; see also Supplemental Material, Table S2). Previous work suggests that this difference is not due to weatherization of homes (Cohen and Gromicko 1988); it may occur because cities are sited in low-lying, alluvial sites, where radon levels are low (Briggs et al. 2008). However, the association persisted after adjustment for geologic unit and community SES. Buildings located in poorer communities also tended to have lower radon concentrations, consistent with past research (Cohen and Gromicko 1988).

We found that buildings using well water had 21% higher indoor radon concentrations than those using municipal water. The release of waterborne radon during showering or washing can contribute to concentrations in buildings. The NRC has estimated that 10,000 pCi/L (37,000 Bq/m^3^) of waterborne radon entering a building is needed to increase indoor air concentration by 1 pCi/L (37 Bq/m^3^) (NRC 1999b). Our 20% increase represented approximately 37 Bq/m^3^. An early study of Pennsylvania groundwater wells reported that only 10% exceeded 185,000 Bq/m^3^ (Swistock et al. 1993), putting our estimate at odds with the rule of thumb.

We found a statistically significant association between proximity to unconventional natural gas wells drilled in the Marcellus shale and first-floor radon concentration in the summer, with a positive—but attenuated—association for basement levels, which suggests a pathway through outdoor ambient air but does not rule out the possibility of radon moving from the basement to the first floor. Geographic location did not appear to account for the association because we did not find an association in buildings prior to 2006 that would be located near Marcellus wells in the future. Prior studies suggest that levels of radon in ambient air are low; our finding merits further study because the Marcellus shale is known to contain elevated levels of uranium (U.S. EPA 2008), and flowback water and reserve pit soil can contain elevated levels of radium, which could create an environmental exposure pathway (Rich and Crosby 2013; Rowan et al. 2011; Warner et al. 2012). It is also possible that radon could enter buildings through the use of natural gas containing radon. However, concentrations at the wellhead in Pennsylvania have a median of 1,369 Bq/m^3^ (Rowan and Kraemer 2012), much lower than the 37,000 Bq/m^3^ thought needed to increase radon concentrations by 12.2 Bq/m^3^ annually in homes that use gas appliances (Gogolak 1980). Our findings should be interpreted in the context of Pennsylvania’s recent *Technologically Enhanced Naturally Occurring Radioactive Material (TENORM)* study report from January 2015, which concluded that

There is little potential for additional radon exposure to the public due to the use of natural gas extracted from geologic formations in Pennsylvania. (Perma-Fix Environmental Services Inc. 2015)

However, the study did detect radon in several components of the unconventional natural gas development process and waste stream, such as natural gas, drill cuttings, and wastewater.

Our analysis had several limitations. We had no information on radon-resistant construction, construction year, types of remediation completed, type of heating and cooking systems, quantity of natural gas and water used in the building, degree of sealing of the building for energy efficiency, soil type near the building, wind speed and direction, or individual SES. These missing data make attributing increased radon levels to a particular source difficult. For instance, it is possible that the observed upward trend from 2004 to 2012 was simply the result of buildings being sealed more tightly during this time.

We did not know whether a radon professional or a homeowner performed each radon test. However, homes are usually tested during real estate transactions, and radon professionals generally perform these tests, ensuring impartial results. Tests are also performed when people are worried about their levels or want to retest after abatement. Worry about levels could introduce a form of selection bias sometimes observed in universal screening programs in which those with higher radon levels would be more likely to test first, which would account for the temporal trends up to 2005. We addressed the abatement concern by only including first measurements. In addition, our analysis should be considered exploratory because we did not perform any environmental radon measurements specifically directed at evaluating the Marcellus or well water hypotheses.

## Conclusion

Radon continues to be a concern in Pennsylvania, and geology is an important contributor. Well water may contribute more to indoor radon than previously thought. There has also been a general rise in concentrations since 2006. The measurements of the Pennsylvania TENORM study should be periodically repeated given the projection of 60,000 wells in Pennsylvania by 2030 (Johnson 2010). Future studies of building radon levels should include more information about buildings, such as age, heating systems, remediation intervention, and radon-resistant construction. Radon exposure represents a major environmental health risk, and in addition to future studies to understand the impact of drilling on radon levels, there is continuing need for a radon program in Pennsylvania to track and evaluate radon concentrations and to encourage testing and remediation.

## Supplemental Material

(343 KB) PDF
