# Supplementary material for: Predictors of Indoor Radon Concentrations in Pennsylvania, 1989–2013
Source: Environ Health Perspect. 2015 Apr 9;123(11):1130–7. doi: 10.1289/ehp.1409014 (PMC4629742; doi:10.1289/ehp.1409014)
Supplement: (343 KB) PDF [file ehp.1409014.s001.acco.pdf]

Environ Health Perspect
doi: 10.1289/ehp.1409014

Note to Readers: *EHP* strives to ensure that all journal content is accessible to all readers. However, some figures and Supplemental Material published in *EHP* articles may not conform to 508 standards due to the complexity of the information being presented. If you need assistance accessing journal content, please contact ehp508@niehs.nih.gov. Our staff will work with you to assess and meet your accessibility needs within 3 working days.

Supplemental Material

Predictors of Indoor Radon Concentrations in Pennsylvania, 1989–2013

Joan A. Casey, Elizabeth L. Ogburn, Sara G. Rasmussen, Jennifer K. Irving, Jonathan Pollak, Paul A. Locke, and Brian S. Schwartz

Table of Contents

Table S1. Number of radon values per year by county category.

Table S2. Predictors and 95% confidence intervals of *ln*-transformed basement and first floor radon concentrations from a linear regression model fit using generalized estimating equations, 1989-2013.

Table S3. Predictors and 95% confidence intervals of *ln*-transformed basement radon concentrations from a linear regression model fit using generalized estimating equations including measurements from up to 4 test dates per building, 1989-2013.

Table S4. Predictors and 95% confidence intervals of *ln*-transformed basement radon concentrations from a linear regression model fit using generalized estimating equations including measurements from up to 4 test dates per building when the first basement radon test at the building was very high (i.e., ≥740 Bq/m^3), 1989-2013.

Table S1. Number of radon values per year by county category.

	County category											
**Year**	**No Marcellus activity**		**Low Marcellus activity**		**High Marcellus activity**		**Reading Prong**		**Philadelphia**		**Total**	
	N	%	N	%	N	%	N	%	N	%	N	%
1989	11,268	59.8	4,397	23.3	824	4.4	1,590	8.4	775	4.1	18,854	100
1990	10,121	57.9	4,294	24.5	737	4.2	1,522	8.7	821	4.7	17,495	100
1991	11,909	61.7	4,492	23.3	634	3.3	1,336	6.9	943	4.9	19,314	100
1992	14,815	65.0	4,550	20.0	608	2.7	1,530	6.7	1291	5.7	22,794	100
1993	15,618	63.8	4,658	19.0	747	3.1	2,090	8.5	1363	5.6	24,476	100
1994	15,560	62.3	4,427	17.7	1055	4.2	2,515	10.1	1412	5.7	24,969	100
1995	17,282	61.8	4,984	17.8	1885	6.7	2,389	8.5	1432	5.1	27,972	100
1996	18,139	59.4	6,307	20.6	2213	7.2	2,398	7.8	1505	4.9	30,562	100
1997	19,798	62.8	6,251	19.8	1769	5.6	2,439	7.7	1256	4.0	31,513	100
1998	24,558	63.5	7,364	19.0	2640	6.8	2,704	7.0	1412	3.7	38,678	100
1999	21,373	60.5	7,472	21.2	2398	6.8	2,595	7.3	1480	4.2	35,318	100
2000	20,138	59.0	7,722	22.6	2389	7.0	2,565	7.5	1305	3.8	34,119	100
2001	20,145	57.6	8,643	24.7	2633	7.5	2,322	6.6	1252	3.6	34,995	100
2002	22,344	60.9	7,743	21.1	2325	6.3	2,663	7.3	1617	4.4	36,692	100
2003	22,438	58.0	8,671	22.4	2645	6.8	3,034	7.8	1910	4.9	38,698	100
2004	23,501	55.7	10,064	23.9	3403	8.1	3,379	8.0	1849	4.4	42,196	100
2005	26,064	53.9	12,166	25.1	3781	7.8	4,187	8.7	2190	4.5	48,388	100
2006	23,925	53.5	11,189	25.0	3413	7.6	3,826	8.6	2381	5.3	44,734	100
2007	24,610	52.9	10,901	23.4	4002	8.6	4,612	9.9	2435	5.2	46,560	100
2008	18,096	52.0	8,560	24.6	3034	8.7	3,220	9.3	1884	5.4	34,794	100
2009	21,774	49.6	10,846	24.7	3976	9.1	4,888	11.1	2413	5.5	43,897	100
2010	23,385	53.6	10,275	23.6	4126	9.5	3,628	8.3	2213	5.1	43,627	100
2011	22,122	53.1	10,035	24.1	4108	9.9	3,339	8.0	2028	4.9	41,632	100
2012	21,335	49.9	10,579	24.8	4264	10.0	3,955	9.3	2584	6.0	42,717	100
2013	21,157	50.7	10,360	24.8	3958	9.5	3,541	8.5	2725	6.5	41,741	100
**Total**	491,475	56.7	196,950	22.7	63567	7.3	72,267	8.3	42476	4.9	866,735	100

Table S2. Predictors and 95% confidence intervals of *ln*-transformed basement and first floor radon concentrations from a linear regression model fit using generalized estimating equations, 1989-2013.

Variable	Model 1A[a] Coefficient (95% CI)	N
**Year**		
1989	0.054 (0.034, 0.074)	18,854
1990	-0.035 (-0.056, -0.015)	17,495
1991	0.037 (0.017, 0.058)	19,314
1992	-0.013 (-0.033, 0.006)	22,794
1993	-0.006 (-0.025, 0.013)	24,476
1994	0.036 (0.018, 0.055)	24,969
1995	0.120 (0.102, 0.137)	27,972
1996	0.071 (0.054, 0.088)	30,562
1997	0.048 (0.032, 0.065)	31,513
1998	0.101 (0.085, 0.117)	38,678
1999	-0.003 (-0.019, 0.013)	35,318
2000	Reference	34,119
2001	0.021 (0.005, 0.037)	34,995
2002	0.003 (-0.013, 0.019)	36,692
2003	-0.067 (-0.082, -0.051)	38,698
2004	-0.057 (-0.072, -0.042)	42,196
2005	-0.019 (-0.033, -0.004)	48,388
2006	0.027 (0.012, 0.042)	44,734
2007	0.040 (0.025, 0.055)	46,560
2008	0.046 (0.030, 0.062)	34,794
2009	0.103 (0.088, 0.118)	43,897
2010	0.070 (0.055, 0.085)	43,627
2011	0.083 (0.068, 0.099)	41,632
2012	0.122 (0.107, 0.138)	42,717
2013	0.073 (0.058, 0.088)	41,741
**Test location**		
First floor	Reference	160,937
Basement	0.655 (0.651, 0.660)	705,798
**Water source**		
Municipal	Reference	730,369
Well water	0.191 (0.184, 0.198)	136,366
**Building type**		
2-Story	Reference	372,012
3-Story	0.160 (0.151, 0.169)	77,845
Apartment	-0.313 (-0.351, -0.276)	3,041
Bi-level	-0.082 (-0.099, -0.065)	15,227
Cape cod	0.057 (0.041, 0.073)	19,638
Commercial	-0.254 (-0.295, -0.212)	2,644
Contemporary	0.050 (0.022, 0.077)	6,124
Public/school	-0.211 (-0.303, -0.120)	572
Ranch	0.140 (0.132, 0.149)	78,710
Split level	-0.075 (-0.089, -0.060)	23,610
Townhouse	-0.376 (-0.386, -0.366)	59,611
Trailer	-0.542 (-0.655, -0.429)	322

Variable	Model 1A[a] Coefficient (95% CI)	N
Unknown	-0.130 (-0.137, -0.123)	207,379
**Test type**		
Activated charcoal	Reference	292,889
Alpha track	0.242 (0.209, 0.276)	8,918
Charcoal liquid scintillation	0.206 (0.195, 0.216)	49,870
Continuous	0.006 (-0.001, 0.013)	224,641
Electret ion chamber	0.125 (0.118, 0.133)	290,417
**Test duration**	-0.0001 (-0.0003, 0.0000)	866,735
**Season**		
Spring	Reference	244,917
Summer	0.058 (0.004, 12.88)	214,327
Autumn	0.156 (0.004, 43.67)	199,684
Winter	0.077 (0.005, 15.4)	207,807
**Average regional temperature (°F)**	0.0043 (0.0035, 0.0050)	866,735
**Average regional temperature^2**	-0.0009 (-0.0010, -0.0009)	866,735
**Average regional temperature^3**	-0.0001 (-0.0001, -0.0001)	866,735
**Average regional rainfall (in)**	-0.0036 (-0.0042, -0.0030)	866,735
**Average regional rainfall^2**	0.0005 (0.0004, 0.0006)	866,735
**Average region rainfall^3**	-0.00001 (-0.00001, -0.00001)	866,735
**Minor civil division**		
Township	Reference	604,479
Borough	-0.132 (-0.139, -0.126)	159,633
City	-0.323 (-0.333, -0.314)	102,621
**County**		
Allegheny	Reference	99,590
Adams	0.582 (0.500, 0.665)	5,689
Armstrong	0.372 (0.318, 0.427)	1,451
Beaver	0.314 (0.289, 0.338)	9,380
Bedford	0.342 (0.262, 0.422)	1,427
Berks	0.749 (0.672, 0.827)	24,192
Blair	0.167 (0.101, 0.233)	5,748
Bradford	-0.050 (-0.134, 0.034)	1,776
Bucks	0.466 (0.388, 0.544)	73,765
Butler	0.292 (0.270, 0.314)	13,519
Cambria	0.053 (0.024, 0.082)	5,688
Cameron	0.247 (0.046, 0.447)	112
Carbon	0.206 (0.139, 0.273)	3,521
Centre	0.281 (0.210, 0.352)	12,264
Chester	0.315 (0.237, 0.393)	70,735
Clarion	0.351 (0.288, 0.414)	1,355
Clearfield	0.470 (0.431, 0.509)	3,170
Clinton	0.289 (0.209, 0.370)	1,425
Columbia	0.541 (0.473, 0.609)	3,447
Crawford	0.114 (0.027, 0.200)	2,090
Cumberland	0.704 (0.627, 0.781)	22,335
Dauphin	0.651 (0.578, 0.725)	20,671
Delaware	0.147 (0.068, 0.225)	52,677
Elk	0.653 (0.577, 0.729)	998
Erie	-0.146 (-0.252, -0.040)	16,785
Fayette	-0.095 (-0.139, -0.051)	2,126

Variable	Model 1A[a] Coefficient (95% CI)	N
Forest	0.497 (0.228, 0.765)	59
Franklin	0.675 (0.595, 0.756)	6,897
Fulton	0.551 (0.406, 0.696)	231
Greene	-0.089 (-0.194, 0.015)	387
Huntingdon	0.296 (0.208, 0.383)	1,168
Indiana	0.153 (0.113, 0.193)	2,746
Jefferson	0.407 (0.345, 0.469)	1,191
Juniata	0.639 (0.544, 0.734)	743
Lackawanna	-0.062 (-0.119, -0.006)	12,753
Lancaster	0.939 (0.861, 1.017)	30,210
Lawrence	0.235 (0.186, 0.284)	2,766
Lebanon	1.129 (1.049, 1.208)	7,091
Lehigh	0.610 (0.532, 0.689)	24,637
Luzerne	-0.077 (-0.132, -0.023)	20,822
Lycoming	0.417 (0.354, 0.481)	6,064
McKean	-0.078 (-0.191, 0.034)	505
Mercer	0.147 (0.091, 0.202)	3,342
Mifflin	0.590 (0.508, 0.671)	1,312
Monroe	-0.301 (-0.368, -0.235)	10,934
Montgomery	0.341 (0.263, 0.418)	113,871
Montour	0.383 (0.303, 0.463)	1,501
Northampton	0.521 (0.443, 0.600)	23,438
Northumberland	0.438 (0.373, 0.503)	4,165
Perry	0.552 (0.478, 0.625)	2,032
Philadelphia	0.363 (0.284, 0.443)	42,476
Pike	-0.460 (-0.529, -0.391)	7,775
Potter	0.318 (0.225, 0.411)	728
Schuylkill	0.599 (0.537, 0.660)	5,409
Snyder	0.472 (0.398, 0.545)	2,022
Somerset	0.068 (0.021, 0.115)	1,999
Sullivan	-0.265 (-0.442, -0.088)	148
Susquehanna	-0.233 (-0.313, -0.154)	1,278
Tioga	-0.434 (-0.518, -0.350)	1,346
Union	0.508 (0.436, 0.580)	3,102
Venango	0.490 (0.423, 0.556)	1,771
Warren	0.441 (0.327, 0.555)	1,405
Washington	0.016 (-0.008, 0.041)	10,347
Wayne	-0.459 (-0.528, -0.390)	4,875
Westmoreland	0.106 (0.089, 0.122)	19,594
Wyoming	-0.062 (-0.146, 0.021)	1,084
York	0.915 (0.837, 0.993)	26,575
**Geologic formation name[b]**		
Stockton formation[c]	Reference	62,026
Primary lithology: Albite-chlorite schist		
Octoraro Formation	0.613 (0.592, 0.634)	18071
Primary lithology: Andesite		
Jonestown Volcanic Suite	0.178 (-0.212, 0.568)	27
Primary lithology: Anorthosite		
Anorthosite	-0.280 (-0.349, -0.212)	991
Anorthosite	-0.025 (-0.132, 0.083)	389

Variable	Model 1A[a]<br>Coefficient (95% CI)	N
Primary lithology: Argillaceous dolomite		
Vintage Formation	0.335 (0.289, 0.380)	2412
Primary lithology: Argillaceous limestone		
Chambersburg Formation	0.538 (0.455, 0.620)	686
Hershey and Myerstown Formations, undivided	0.354 (0.272, 0.436)	663
Hershey Formation through Annville Formation, undivided	0.805 (0.478, 1.132)	38
Limestone of Martinsburg Formation	0.378 (0.328, 0.428)	2433
Primary lithology: Argillaceous shale		
Girard Shale	0.573 (0.453, 0.692)	8147
Primary lithology: Argillite		
Lockatong Formation	0.147 (0.130, 0.164)	24068
Primary lithology: Arkosic sandstone		
New Oxford Formation	-0.050 (-0.084, -0.017)	5646
Sedimentary strata at Jacksonwald and Aspers	0.178 (-0.036, 0.392)	89
Primary lithology: Black shale		
Marcellus Formation	1.078 (0.996, 1.159)	1960
Primary lithology: Calcareous sandstone		
Decker Formation through Poxono Island Formation, undivided	0.654 (0.461, 0.846)	127
Primary lithology: Calcareous shale		
Elbrook Formation	0.257 (0.227, 0.287)	6798
Onondaga and Old Port Formations, undivided	0.605 (0.529, 0.680)	1967
Onondaga Formation	0.660 (0.337, 0.982)	42
Wills Creek Formation	0.309 (0.239, 0.379)	3545
Primary lithology: Diabase		
Peters Creek Schist	0.652 (0.601, 0.703)	1840
Primary lithology:		
Diabase	-0.297 (-0.331, -0.263)	4450
Primary lithology: Dolomite		
Allentown Formation	0.414 (0.385, 0.443)	23089
Bellefonte and Axemann Formations, undivided	0.464 (0.301, 0.626)	159
Bellefonte Formation	1.257 (1.172, 1.342)	2788
Gatesburg Formation	0.934 (0.781, 1.087)	210
Ledger Formation	0.246 (0.217, 0.276)	6733
Leithsville Formation	0.329 (0.291, 0.367)	4725
Lower Cambrian rocks, undivided	0.705 (0.634, 0.775)	924
Mines Member of Gatesburg Formation	1.071 (0.953, 1.189)	567
Nittany and Stonehenge/Larke Formations, undivided	1.295 (1.158, 1.432)	282
Nittany Formation	1.169 (1.078, 1.260)	2056
Ontelaunee Formation	0.666 (0.621, 0.712)	2582
Pinesburg Station Formation	0.670 (0.598, 0.741)	963
Richland Formation	0.509 (0.461, 0.556)	2390
Rickenbach Formation	0.438 (0.389, 0.486)	2411
Snitz Creek Formation	0.505 (0.407, 0.604)	440
Tomstown Formation	0.206 (0.144, 0.268)	1306
Zooks Corner Formation	0.387 (0.346, 0.428)	3360
Primary lithology: Feldspathic quartz sand		
Pensauken and Bridgeton Formations, undifferentiated	-0.152 (-0.171, -0.133)	23651
Primary lithology: Feldspathic quartzite		
Setters Quartzite	0.200 (0.118, 0.282)	703

Variable	Model 1A[a] Coefficient (95% CI)	N
Primary lithology: Felsic gneiss		
Felsic and intermediate gneiss	0.220 (0.190, 0.251)	6644
Felsic and intermediate gneiss	-0.035 (-0.063, -0.006)	7420
Felsic gneiss	-0.175 (-0.193, -0.156)	19922
Felsic to mafic gneiss	0.600 (0.561, 0.640)	3692
Primary lithology: Ferruginous clay		
Patapsco Formation	0.129 (0.046, 0.213)	677
Primary lithology: Granitic gneiss		
Granitic gneiss and granite	0.130 (0.103, 0.158)	7905
Primary lithology: Granitic pegmatite		
Pegmatite	0.008 (-0.167, 0.184)	151
Primary lithology: Graphitic felsic gneiss		
Graphitic felsic gneiss	0.307 (0.247, 0.368)	1315
Primary lithology: Graphitic gneiss		
Graphitic felsic gneiss	0.252 (0.213, 0.290)	3476
Primary lithology: Graphitic marble		
Franklin Marble	1.607 (1.452, 1.762)	197
Primary lithology: Gravelly sand		
Bryn Mawr Formation	-0.089 (-0.120, -0.057)	6169
Trenton Gravel	-0.192 (-0.215, -0.168)	15202
Primary lithology: Graywacke		
Graywacke and shale of Martinsburg Formation	0.705 (0.659, 0.750)	3037
Graywacke of Hamburg sequence	0.458 (0.406, 0.510)	2126
Primary lithology: Greenstone schist		
Greenstone schist	0.323 (0.064, 0.581)	60
Primary lithology: High-calcium limestone		
Annville Formation	1.158 (0.986, 1.329)	159
Primary lithology: Limestone		
Axemann Formation	1.261 (1.166, 1.356)	1507
Beekmantown Group	0.263 (0.212, 0.315)	2118
Benner Formation through Loysburg Formation, undivided	1.063 (0.961, 1.165)	885
Buffalo Springs Formation	0.284 (0.236, 0.332)	2207
Coburn Formation through Loysburg Formation, undivided	0.671 (0.529, 0.814)	262
Coburn Formation through Nealmont Formation, undivided	0.624 (0.510, 0.739)	540
Conestoga Formation	0.116 (0.095, 0.138)	18466
Epler Formation	0.568 (0.540, 0.597)	11527
Keyser and Tonoloway Formations, undivided	0.585 (0.517, 0.654)	5139
Keyser Formation through Clinton Group, undivided	0.355 (-0.249, 0.960)	11
Keyser Formation through Mifflintown Formation, undivided	0.349 (0.214, 0.484)	314
Limestone of Hamburg sequence	0.383 (0.314, 0.453)	1015
Millbach and Schaefferstown Formations, undivided	0.495 (0.360, 0.630)	237
Millbach Formation	0.333 (0.277, 0.388)	1556
Monongahela Group	0.505 (0.426, 0.583)	42447
Rockdale Run Formation	0.769 (0.726, 0.811)	4193
Shadygrove Formation	0.763 (0.681, 0.846)	708
Shriver, Mandata, Corriganville, and New Creek Members of Old Port Formation, undivided	0.851 (-0.043, 1.745)	5
Snitz Creek and Buffalo Springs Formations, undivided	0.479 (0.390, 0.568)	559
St. Paul Group	0.709 (0.667, 0.751)	4074
Stonehenge Formation	0.703 (0.659, 0.746)	2913

Variable	Model 1A[a] Coefficient (95% CI)	N
Stonehenge/Larke Formation	1.386 (1.288, 1.485)	1245
Warrior Formation	0.694 (0.451, 0.936)	75
Zullinger Formation	0.623 (0.564, 0.682)	1558
Primary lithology: Limestone conglomerate		
Limestone fanglomerate	0.328 (0.252, 0.404)	771
Primary lithology: Mafic gneiss		
Banded mafic gneiss	0.378 (0.317, 0.438)	1274
Hornblende gneiss	0.321 (0.252, 0.390)	1006
Mafic gneiss	-0.008 (-0.055, 0.040)	2212
Mafic gneiss	-0.243 (-0.298, -0.189)	1678
Mafic gneiss	-0.104 (-0.157, -0.052)	1857
Mafic gneiss	-0.188 (-0.236, -0.139)	2235
Primary lithology: Marble		
Cockeysville Marble	0.234 (0.112, 0.356)	309
Primary lithology: Metabasalt		
Metabasalt	-0.322 (-0.440, -0.203)	307
Sams Creek Metabasalt	0.266 (0.117, 0.415)	187
Primary lithology: Metadiabase		
Metadiabase	0.172 (0.044, 0.300)	292
Primary lithology: Metarhyolite		
Metarhyolite	0.600 (0.507, 0.693)	510
Primary lithology: Mudstone		
Brunswick Formation	0.026 (0.012, 0.040)	48260
Lock Haven Formation	0.900 (0.823, 0.978)	3685
Sherman Creek Member of Catskill Formation	0.917 (0.850, 0.983)	2420
Primary lithology: Oligoclase-mica schist		
Glenarm Wissahickon formation	0.096 (0.073, 0.120)	13493
Wissahickon Formation	0.120 (0.104, 0.136)	57742
Primary lithology: Phyllite		
Harpers Formation	0.087 (-0.004, 0.177)	531
Marburg Schist	0.756 (0.715, 0.797)	3422
Primary lithology: Quartz conglomerate		
Gettysburg conglomerate	0.028 (-0.043, 0.099)	910
Hammer Creek conglomerate	-0.067 (-0.124, -0.009)	1432
New Oxford conglomerate	-0.021 (-0.173, 0.130)	178
Quartz fanglomerate	0.098 (0.030, 0.166)	1027
Stockton conglomerate	0.082 (0.000, 0.165)	758
Primary lithology: Quartzite		
Antietam and Harpers Formations, undivided	0.525 (0.491, 0.558)	5517
Antietam Formation	0.448 (0.389, 0.507)	1342
Chickies Formation	0.129 (0.104, 0.153)	10112
Hardyston Formation	0.322 (0.266, 0.378)	1612
Montalto Member of Harpers Formation	-0.199 (-0.349, -0.050)	195
Tuscarora Formation	0.466 (0.268, 0.663)	113
Weverton and Loudoun Formations, undivided	-0.312 (-0.569, -0.055)	62
Primary lithology: Sandstone		
Allegheny Formation	0.371 (0.296, 0.447)	11226
Bald Eagle Formation	0.584 (0.335, 0.834)	70
Berea Sandstone through Riceville Formation, undivided	0.271 (0.135, 0.408)	507
Berea Sandstone through Venango Formation, undivided	0.212 (0.082, 0.342)	547

Variable	Model 1A[a] Coefficient (95% CI)	N
Burgoon Sandstone	0.716 (0.522, 0.910)	124
Burgoon Sandstone through Cuyahoga Group, undifferentiated	0.339 (0.050, 0.628)	54
Catskill Formation	0.596 (0.534, 0.659)	12168
Clarks Ferry Member of Catskill Formation	1.475 (1.020, 1.931)	20
Corry Sandstone through Riceville Formation, undivided	0.384 (0.227, 0.540)	259
Duncannon Member of Catskill Formation	0.337 (0.270, 0.405)	3858
Foreknobs Formation	0.546 (0.377, 0.716)	167
Greene Formation	0.747 (0.629, 0.865)	561
Hammer Creek Formation	-0.205 (-0.253, -0.158)	2194
Huntley Mountain Formation	0.803 (0.653, 0.953)	230
Juniata Formation	0.362 (0.110, 0.614)	68
Llewellyn Formation	0.330 (0.270, 0.391)	20309
Long Run and Walcksville Members of Catskill Formation, undivided	0.781 (0.709, 0.852)	7272
Long Run Member of Catskill Formation	0.991 (0.913, 1.068)	2123
Lower members of Gatesburg Formation, undivided	0.920 (0.829, 1.011)	2124
Packerton Member of Catskill Formation	0.643 (0.482, 0.803)	199
Pocono Formation	0.375 (0.246, 0.503)	303
Poplar Gap and Packerton Members of Catskill Formation, undivided	0.551 (0.476, 0.626)	3362
Poplar Gap Member of Catskill Formation	0.370 (0.290, 0.450)	2019
Pottsville Formation	0.373 (0.304, 0.442)	9077
Ridgeley Formation through Coeymans Formation, undivided	0.926 (0.774, 1.078)	221
Ridgeley Member of Old Port Formation	0.536 (0.344, 0.728)	141
Shawangunk Formation	0.459 (0.052, 0.866)	25
Shenango Formation	0.269 (0.165, 0.372)	849
Shenango Formation through Oswayo Formation, undivided	-0.119 (-0.274, 0.037)	251
Shenango Formation through Riceville Formation, undivided	0.341 (0.036, 0.647)	54
Spechty Kopf Formation	0.512 (0.344, 0.680)	161
Towamensing Member of Catskill Formation	0.929 (0.841, 1.017)	1244
Walcksville and Towamensing Members of Catskill Formation, undivided	1.004 (0.806, 1.203)	117
Walcksville Member of Catskill Formation	1.210 (1.111, 1.309)	752
Washington Formation	0.430 (0.335, 0.525)	1694
Waynesburg Formation	0.493 (0.412, 0.573)	11979
Primary lithology: Serpentinite		
Ultramafic rocks	0.166 (0.117, 0.215)	2094
Primary lithology: Shale		
Bloomsburg and Mifflintown Formations, undivided	0.284 (0.210, 0.358)	2500
Bloomsburg Formation	0.482 (0.365, 0.599)	392
Casselman Formation	0.734 (0.656, 0.812)	55531
Clinton Group	0.376 (0.298, 0.454)	1804
Cocalico Formation	0.807 (0.764, 0.849)	3065
Glenshaw Formation	0.611 (0.534, 0.689)	49486
Hamburg sequence rocks	0.682 (0.653, 0.712)	18260
Hamilton Group	0.431 (0.367, 0.495)	7345
Heidlersburg Member of Gettysburg Formation	0.117 (0.028, 0.207)	594
Kinzers Formation	0.398 (0.350, 0.447)	2076
Mahantango Formation	1.005 (0.927, 1.082)	2338
Martinsburg Formation	0.682 (0.651, 0.713)	11148
Mauch Chunk Formation	0.663 (0.607, 0.720)	5632
Northeast Shale	0.519 (0.398, 0.640)	4263

Variable	Model 1A[a] Coefficient (95% CI)	N
Onondaga Formation through Poxono Island Formation, undivided	0.496 (0.331, 0.660)	183
Reedsville Formation	0.713 (0.589, 0.837)	375
Shale and graywacke of Hamburg sequence	0.899 (0.842, 0.956)	1524
Waynesboro Formation	0.350 (0.267, 0.433)	698
Primary lithology: Shaly limestone		
Jacksonburg Formation	0.235 (0.186, 0.283)	2493
Primary lithology: Siliceous sandstone		
Buttermilk Falls Limestone through Esopus Formation, undivided	1.026 (0.929, 1.123)	866
Primary lithology: Siltstone		
Beaverdam Run Member of Catskill Formation	1.373 (1.253, 1.493)	381
Brallier and Harrell Formations, undivided	0.574 (0.501, 0.648)	2964
Buddys Run Member of Catskill Formation	0.767 (0.624, 0.909)	252
Chadakoin Formation	0.320 (0.203, 0.436)	4003
Cuyahoga Group	0.318 (0.225, 0.411)	1909
Irish Valley Member of Catskill Formation	0.947 (0.877, 1.017)	2105
Scherr Formation	0.516 (0.339, 0.693)	149
Trimmers Rock Formation	0.941 (0.875, 1.006)	4360
Venango Formation	0.541 (0.419, 0.663)	1814
Primary lithology: Silty mudstone		
Gettysburg Formation	-0.063 (-0.095, -0.030)	6772
Primary lithology: Slate		
Peach Bottom Slate and Cardiff Conglomerate, undivided	0.845 (0.534, 1.155)	42
**Intercept**	3.493 (3.414, 3.573)	866,735

[a]Model 1A was adjusted for: test year (1989-2013); test location (basement or first floor); well water use (yes or no); 13 building types (including "unknown"); test type (activated charcoal, alpha-track detectors, charcoal liquid scintillation, continuous radon monitors, and electret ion chamber); test duration; season (Spring: March-May, Summer: June-August, Fall: September-November, and Winter: December-February); weather (average temperature and rainfall for 10 regions during month radon measurement began with linear, quadratic and cubic terms to account for nonlinearity); minor civil division (township, borough, or city); county (n = 67); and 179 mutually exclusive geologic units (reference group = Stockton Formation [n = 62,026] and 12 geologic units with less than 20 measurements). [b]Geologic unit names from Pennsylvania Geological Survey, 2001. [c]The reference group for geologic unit contained observations from the Stockton Formation (n = 62,026) and 12 geologic units with <20 observations: the Shenango Formation through Cuyahoga Group, undivided (n = 19); the Conemaugh Group (n = 15); the Valentine Member of Benner Formation (n = 15); the Keyser Formation through Clinton Group, undivided (n = 11); the Allegheny and Pottsville Formations, undivided (n = 6); the Berry Run and Sawmill Run Members of Catskill Formation, undivided (n = 5); the Shriver, Mandata, Corriganville, and New Creek Members of Old Port Formation, undivided (n = 5); the Wills Creek Formation through Mifflintown Formation, undivided (n = 4); the Metagabbro (n = 3); the Juniata and Bald Eagle Formations, undivided (n = 2); the Sands of Presque Isle (n = 2); and the Wakefield Marble (n = 1).

Table S3. Predictors and 95% confidence intervals of *ln*-transformed basement radon concentrations from a linear regression model fit using generalized estimating equations including measurements from up to 4 test dates per building, 1989-2013.

Variable	Model 1B[a]<br>Coefficient (95% CI)	N
**Test number**		
1	Reference	713,030
2	-0.463 (-0.467, -0.458)	212,378
3	-0.724 (-0.732, -0.716)	67,049
4	-0.867 (-0.880, -0.854)	23,307
**Intercept**	4.106 (4.024, 4.189)	1,015,764

[a]Model 1B was adjusted for: test year (1989-2013); well water use (yes or no); 13 building types (including "unknown"); test type (activated charcoal, alpha-track detectors, charcoal liquid scintillation, continuous radon monitors, and electret ion chamber); test duration; season (Spring: March-May, Summer: June-August, Fall: September-November, and Winter: December-February); weather (average temperature and rainfall for 10 regions during month radon measurement began with linear, quadratic and cubic terms to account for nonlinearity); minor civil division (township, borough, or city); county (n = 67); and 179 mutually exclusive geologic units (reference group = Stockton Formation [n = 62,026] and 11 geologic units with less than 20 measurements: Valentine Member of Benner Formation [n = 18]; Keyser Formation through Clinton Group, undivided [n = 16]; Conemaugh Group [n = 16]; Berry Run and Sawmill Run Members of Catskill Formation, undivided [n = 9]; Shriver, Mandata, Corriganville, and New Creek Members of Old Port Formation, undivided [n = 6]; Wills Creek Formation through Mifflintown Formation, undivided [n = 6]; Allegheny and Pottsville Formations, undivided [n = 4]; Juniata and Bald Eagle Formations, undivided [n = 3]; Sands of Presque Isle [n = 2]; Metagabbro [n = 2]; and Wakefield Marble [n = 1]).

Table S4. Predictors and 95% confidence intervals of *ln*-transformed basement radon concentrations from a linear regression model fit using generalized estimating equations including measurements from up to 4 test dates per building when the first basement radon test at the building was very high (i.e., ≥740 Bq/m^3), 1989-2013.

Variable	Model 1B[a] Coefficient (95% CI)	N
**Test number**		
1	Reference	55,123
2	-2.185 (-2.200, -2.170)	28,342
3	-2.561 (-2.583, -2.538)	11,093
4	-2.656 (-2.688, -2.623)	4,662
**Intercept**	7.053 (6.792, 7.314)	99,220

[a]The restricted model 1B was adjusted for: test year (1989-2013); well water use (yes or no); 13 building types (including "unknown"); test type (activated charcoal, alpha-track detectors, charcoal liquid scintillation, continuous radon monitors, and electret ion chamber); test duration; season (Spring: March-May, Summer: June-August, Fall: September-November, and Winter: December-February); weather (average temperature and rainfall for 10 regions during month radon measurement began with linear, quadratic and cubic terms to account for nonlinearity); minor civil division (township, borough, or city); county (n = 67); and 179 mutually exclusive geologic units (reference group = Stockton Formation [n = 62,026] and the 11 geologic units with less than 20 measurements in model 1B).
